# Virological Outcomes and Drug Resistance in Chinese Patients after 12 Months of 3TC-Based First-Line Antiretroviral Treatment, 2011–2012

**DOI:** 10.1371/journal.pone.0088305

**Published:** 2014-02-07

**Authors:** Jing Wang, Cui He, Jenny H. Hsi, Xiaoqin Xu, Yong Liu, Jianmei He, Hua Ling, Ping Ding, Yi Tong, Xiaobai Zou, Quanhua Zhou, Lingjie Liao, Xia Wang, Yuhua Ruan, Yiming Shao, Hui Xing

**Affiliations:** 1 State Key Laboratory for Infectious Disease Prevention and Control, National Center for AIDS/STD Control and Prevention, Chinese Center for Disease Control and Prevention, Collaborative Innovation Center for Diagnosis and Treatment of Infectious Diseases, Beijing, China; 2 Jiangsu Center for Disease Control and Prevention, Nanjing, China; 3 Guizhou Center for Disease Control and Prevention, Guiyang, China; 4 Hunan Center for Disease Control and Prevention, Changsha, China; 5 Chongqing Center for Disease Control and Prevention, Chongqing, China; University of Pittsburgh, United States of America

## Abstract

**Objective:**

To determine the prevalence of virological failure and HIV drug resistance among Chinese patients one year after initiating lamivudine-based first-line antiretroviral treatment.

**Methods:**

A prospective cohort study with follow-up at 12 months was conducted in four urban sentinel sites in China. Antiretroviral naive patients ≥18 years old were recruited. Blood samples were collected for testing CD4 cell count, viral load, and (for samples with HIV-1 RNA ≥1000 copies/ml) genotyping of drug resistance.

**Results:**

A total of 513 patients were enrolled in this cohort, of whom 448 (87.3%) were retained at 12 months. The median final CD4 cell count was 313 cells/mm^3^, which increased from 192 cells/mm^3^ at baseline (P<0.0001). Of the 448 remaining subjects, 394 (87.9%) had successful virological suppression (HIV RNA <1000 copies/ml). Among 54 samples with viral load ≥1000 copies/ml, 40 were successfully genotyped, and 11 were found with detectable HIV drug resistance mutations. Of these, the proportions of drug resistance to NNRTIs, NRTIs and PIs were 100%, 81.8% and 0%, respectively. Injecting drug use (AOR = 0.40, 95% CI: 0.19,0.84; P = 0.0154), CD4 count at baseline ≥350 cells/mm^3^ (AOR = 0.32, 95% CI: 0.14,0.72; P = 0.0056), and missed doses in the past month (AOR = 0.30, 95% CI: 0.15,0.60; P = 0.0006) were significantly negatively associated with HIV RNA <1000 copies/ml.

**Conclusions:**

Our study demonstrates effective virological and immunological outcomes at 12 months among these who initiated first-line ART treatment. However, patients infected through drug injection, who missed doses, or with higher CD4 count at baseline are at increased risk for poor virological response.

## Introduction

The rapid expansion of combination antiretroviral treatment (cART) has dramatically improved the prognosis of patients infected with HIV and decreased HIV/AIDS associated morbidity and mortality [1]–[5]. Since year 2000, most developing countries have initiated cART treatment programs, many of which were scaled-up in 2003 through the “3 by 5” initiative. At the end of 2011, more than 8 million people in low- and middle-income countries were receiving antiretroviral therapy (ART), up from 6.6 million in 2010 which represented an increase of about 20% [6].

Since the China’s National Free Antiretroviral Treatment Program (NFATP) began in 2002, cART use has scaled up rapidly. By the end of 2012, more than 208,216 patients across the country had received free antiretroviral treatment [7]. Many studies found that the NFATP has successfully increased life expectancy and reduced mortality among Chinese HIV patients [8]–[11]. However, although rapid cART scale-up significantly decreased AIDS-related morbidity and mortality, HIV antiretroviral treatment programs are facing the challenge of potential widespread emergence and transmission of HIV drug resistance (HIVDR) [11]–[15].

In 2004, The World Health Organization (WHO), in collaboration with the United States Centers for Disease Control and Prevention (CDC) and through HIVResNet developed a global strategy for the assessment and prevention of HIVDR. The protocol for population-based surveillance of acquired HIVDR at sentinel ART clinics is designed to be used particularly in resource-limited countries, where decisions on ART regimens for a given patient population are made through monitoring HIVDR emergence during treatment in addition to related program factors [16]. In China, based on the WHO HIVDR surveys under the direction of the Ministry of Health HIVDR working group, studies have shown that community-based ART had significant effects on viral suppression and acquired HIVDR at six sentinel sites in China [17]–[18]. However, the NFATP’s first-line antiretroviral regimens have since been changed to exclude the use of didanosine (DDI) and to include the use of lamivudine (3TC) for all new patients initiating treatment [19]. Due to these changes and the rapid scale-up of ART in China, the aim of this prospective survey study is to evaluate HIVDR in a cohort of Chinese HIV patients recently initiating 3TC-based first-line ART at four sentinel sites in 2011–2012.

## Methods

### Study Design and Study Participants

The four sentinel surveys were conducted in urban areas of China, including Jiangsu province (Nanjing, Suzhou, and Nantong cities), Guizhou province (Guiyang, Tongren, and Dujun cities), Hunan province (Hengyang City) and Chongqing Municipality (Shapingba, Jiulongpo, and Yuzhong districts), respectively, where most of HIV infection were transmitted through heterosexual intercourse and homosexual intercourse [7]. The survey was a prospective cohort study with follow-up at 12 months. The study subjects were recruited through sequential sampling at each clinic to participate in a one-year prospective cohort study. Criteria for enrolling in the study were: previously antiretroviral naive, 18 years or older, agreeing to initiate ART in the NFATP, and willing to provide informed consent. A baseline screening survey was conducted in 2011. The national treatment criterion at that time was: (1) CD4 cell count<350/mm^3^; (2) World Health Organization (WHO) stage III/IV diseases, or (3) willingness to receive ART, regardless of the criteria 1 and 2. The ART regimen will be provided through the NFATP. First-line ART regimen consisted of tenofovir (TDF) or azidothymidine (AZT)+lamivudine (3TC)+efavirenz (EFV) or nevirapine(NVP) [19]. The main study outcome was HIVDR emergence after the first year of ART. The specific objectives were to: (1) estimate the proportion of HIV RNA suppression to <1000 copies/ml and associated factors at 12 months after starting first-line ART; and (2) identify the prevalence of specific HIVDR mutations among those with viral load ≥1000 copies/ml [16].

### Ethics Statement

The study was approved by the institutional review board of the National Center for AIDS/STD Control and Prevention of the China Center for Disease Control and Prevention (NCAIDS, China CDC).

### Data Collection

Data were collected using an interviewer-administered questionnaire. Each study participant was assigned a confidential identification number used to label questionnaires and blood specimens. The questionnaire was administered by trained interviewers in a private room. Questionnaires included demographic data, ART treatment and self-reported adherence data. Demographic variables included age, sex, ethnicity, education level, marital status, occupation and HIV transmission route. ART treatment variables included WHO clinical stage, initial regimen, ART distribution institution, ART refill interval, missed ART doses in the past month, ratio of on-time drug intake in the past month, and reasons for loss of treatment retention.

### Laboratory Tests

All subjects provided blood specimens for testing CD4^+^ T-lymphocyte count (CD4 count), HIV viral load, and HIV drug resistance mutations at baseline and 12-month follow-up. CD4 count was tested using flow cytometry (FACSC Calibur, BD Company, USA) within 24 hours after specimen collection in local CDCs. Blood plasma was isolated and stored frozen at −80°C at local CDCs before transferring to NCAIDS in Beijing for testing viral load and drug resistance mutations. Plasma HIV RNA was quantified with real-time NASBA (NucliSense Easy Q, bioMérieux, France) or with COBAS (Roche Applied Biosystems, Germany) according to the manufacturers’ recommendations. For the WHO surveys, successful viral suppression was defined as HIV RNA level <1,000 copies/ml using a quality-assured viral load assay. In samples with viral load ≥1000 copies/ml, HIV drug resistance genotyping was performed at the WHO Accreditation Laboratory of NCAIDS, China CDC by using an in-house method as previously described [17]–[18]. Drug resistance mutation analysis and viral subtype determination were performed on a 1.3 kb section of the HIV *pol* gene using the Stanford University HIV Drug Resistance Database online sequence analysis tool (http://hivdb.stanford.edu/pages/algs/sierra_sequence.html). We included mutation results that conferred low-, intermediate-, and high- level resistance [20]–[22].

### Data Analysis

Questionnaire and laboratory data were double-entered and compared using EpiData software (The EpiData Association Odense, Denmark). Data were then converted and analyzed using Statistical Analysis System version 9.1 (SAS Institute Inc., Cary, NC, USA). The data were analyzed using unadjusted odds ratios with a test for significance according to chi-square test and Fisher’s exact test. Variables that were significantly (p<0.05) associated with HIV RNA <1000 copies/ml at 12 months in the univariable analyses were considered for inclusion in the multivariable logistic regression model. Multivariable logistic regression was performed to examine the independent effect of each factor under consideration. All tests of significance were two-sided, with p-value <0.05 indicating that an association was statistically significant.

## Results

### Demographic Characteristics

Among 535 consecutive participants enrolled, one was less than 18 years old, three refused to participate in the study, five did not receive national free regimen, and 13 patients with baseline HIVDR were excluded from this study. Thus, 513 subjects entered this prospective cohort and provided written informed consent. The baseline and 12-month follow-up characteristics were shown in Table 1. Of 513 eligible patients, 139, 123, 119 and 132 participants were from Jiangsu, Guizhou, Hunan, Chongqing, respectively; 78.6% were male; the mean age were 38.8 years (SD, ±11.2 years); 90.1% belonged to the Han ethnic group; 47.0% had education level of junior high school or lower; 48.2% were infected through heterosexual intercourse; and the most common HIV viral subtype was CRF01_AE.

**Table 1:** Characteristics of HIV patients receiving first-line ART in four sentinel antiretroviral treatment sites.

Characteristics	Jiangsu	Guizhou	Hunan	Chongqing	Total
	N (%)	N (%)	N (%)	N (%)	N (%)
Overall	139	123	119	132	513
Age (mean ± SD, year)	39.7±11.3	34.5±8.6	41.5±10.1	39.4±12.9	38.8±11.2
Sex					
Male	121 (87.1)	85 (69.1)	86 (72.3)	111 (84.1)	403 (78.6)
Female	18 (12.9)	38 (30.9)	33 (27.7)	21 (15.9)	110 (21.4)
Ethnicity					
Han	136 (97.8)	75 (61.0)	119(100.0)	132 (100.0)	462 (90.1)
Other	3 (2.2)	48 (39.0)	0	0	51 (9.9)
Education					
Junior high school or less	65 (46.8)	51 (41.5)	83(69.8)	42 (31.8)	241 (47.0)
High school or more	74 (53.2)	72 (58.5)	36(30.2)	90 (68.2)	272(53.0)
Marital status					
Married	87 (62.6)	55 (44.7)	74 (62.2)	63 (47.7)	279 (54.4)
Other	52 (37.4)	68 (55.3)	45 (37.8)	69 (52.3)	234 (45.6)
Occupation					
Farmer	16 (11.5)	7 (5.7)	21 (17.6)	3 (2.3)	47 (9.2)
Other	123 (88.5)	116 (94.3)	98 (82.4)	129 (97.7)	466 (90.8)
HIV transmission route					
Heterosexual intercourse	67 (48.2)	60 (48.8)	71 (59.7)	49 (37.1)	247 (48.2)
Homosexual intercourse	67 (48.2)	26 (21.1)	3 (2.5)	62 (47.0)	158 (30.8)
Drug injection	0	29 (23.6)	33 (27.7)	12 (9.1)	74 (14.4)
Other	5 (3.6)	8 (6.5)	12 (10.1)	9 (6.8)	34 (6.6)
HIV subtypes					
CRF01_AE	90(64.7)	54(43.9)	88(74.0)	15(11.4)	247(48.1)
CRF08_BC	4(2.9)	3(2.4)	7(5.9)	11(8.3)	25(4.9)
CRF07_BC	24(17.3)	37(30.1)	7(5.9)	99(75.0)	167(32.6)
Other	21(15.1)	29(23.6)	17(14.2)	7(5.3)	74(14.4)
WHO clinic stage III or IV at baseline	33 (23.7)	25 (20.3)	114 (95.8)	26 (19.7)	198 (38.6)
HIV RNA <1000 copies/mL at baseline	7 (5.0)	14 (11.4)	5 (4.2)	4 (3.0)	30 (5.9)
CD4 count at baseline (cells/mm^3^)					
350 or above	4 (2.9)	11 (8.9)	0	30 (22.7)	45 (8.8)
200–349	60 (43.1)	45 (36.6)	21 (17.6)	62 (47.0)	188 (36.7)
100–199	35 (25.2)	36 (29.3)	32 (26.9)	28 (21.2)	131 (25.5)
0–99	40 (28.8)	31 (25.2)	66 (55.5)	12 (9.1)	149 (29.0)
Initial ART regimen					
AZT+3TC+NVP	115 (82.7)	61 (49.6)	26 (21.9)	101 (76.5)	303 (59.1)
D4T+3TC+NVP	10 (7.2)	9 (7.3)	10 (8.4)	4 (3.0)	33 (6.4)
AZT+3TC+EFV	9 (6.5)	34 (27.7)	13 (10.9)	18 (13.7)	74 (14.4)
D4T+3TC+EFV	5 (3.6)	18 (14.6)	21 (17.7)	5 (3.8)	49 (9.5)
3TC+TDF+EFV	0	0	43(36.1)	4(3.0)	47(9.2)
3TC+TDF+NVP	0	1(0.8)	6(5.0)	0	7(1.4)
Retention at 12-month follow-up study	128(92.1)	109(88.6)	94(79.0)	117(88.6)	448(87.3)
Duration of follow-up (median, month)	12.1	12.1	12.1	11.9	12.0
Reasons for not retained at 12 months					
Death	3 (2.2)	6 (4.9)	14 (11.8)	2 (1.5)	25 (4.9)
Transferred out	0	0	1 (0.8)	0	1 (0.2)
Loss to follow-up	1 (0.7)	6 (4.9)	8 (6.7)	10(7.6)	25 (4.9)
Other	7 (5.0)	2 (1.6)	2(1.7)	3(2.3)	14 (2.7)
Missed doses in the past month	12 (9.4)	10 (9.2)	26 (27.7)	15 (12.8)	63 (14.1)
Ratio (95%) of on-time drug intake in the past month	100 (78.1)	92 (84.4)	77 (81.9)	107 (91.5)	376 (83.9)
On first-line ART at 12 months	127(99.2)	109(100.0)	90(95.7)	115(98.3)	441(98.4)
HIV RNA <1000 copies/ml at 12 months	113 (88.3)	102(93.6)	82 (87.2)	97 (82.9)	394 (87.9)
HIV RNA <500 copies/ml at 12 months	113(88.3)	102(93.6)	77(81.9)	86(73.5)	378(84.4)
HIV RNA <200 copies/ml at 12 months	113(88.3)	102(93.6)	76(80.9)	81(69.2)	372(83.0)
HIVDR among HIV RNA ≥1000 copies/ml with PCR products at 12 months	3/12(25.0)	(1/5) (20.0)	(4/8)(50.0)	(3/15)(20.0)	(11/40)(27.5)

### HAART Regimens and Immunologic Profiles

All patients received free standard first-line regimen through the NFATP, and 98.4% maintained the initial regimen at follow-up. Only 7(1.6%) patients switched to the second-line regimen during the follow-up. The initial ART regimen were AZT+3TC+NVP(59.1%), D4T+3TC+NVP(6.4%), AZT+3TC+EFV(14.4%), D4T+3TC+EFV(9.5%), 3TC+TDF+EFV(9.2%) and 3TC+TDF+NVP(1.4%). Among 65 participants who were lost to follow up, 4.9% had died, 4.9% were lost to contact, and 0.2% transferred out of the study districts (Table 1).

Of the 448 patients retained at 12 month, CD4 cell count was 313 cells/mm^3^, which increased from the median 192 cells/mm^3^ at baseline(P<0.0001). 14.1% reported missing doses, and 83.9% maintained ≥95% of on-time drug intake in the past month.

### Genotypic Drug Resistance and Predictors for Viral Suppression Success

After receiving ART for nearly one year, 394(87.9%) patients had plasma HIV RNA <1000 copies/ml. Among the 54 samples with viral load ≥1000 copies/ml, 40 were successfully genotyped. Of the 11 with detectable HIVDR mutations, the proportion of drug resistance to NNRTIs, NRTIs and PIs were 100%, 81.8% and 0%, respectively. Among these, 9(81.8%) patients had multi-drug resistance to NNRTIs and NRTIs. The most common NNRTIs mutation site were K103N(36.4%), Y818C/I/V(27.3%) and K101E(18.2%). As well, M184V/I(72.7%), K65R(27.3%), D67N(27.3%) were found as the most frequent mutations to NRTIs (Table 2).

**Table 2:** HIV drug resistance and drug resistance mutations among the 11 patients with HIVDR mutations detected at 12 months at four sentinel antiretroviral treatment sites.

Antiretroviral drug	N (%)	HIV drug resistance mutation, N (%)
Overall	11 (100.0)	
Non-nucleoside reverse transcriptase inhibitors (NNRTI, any)	11 (100.0)	K101E, 2 (18.2)
Efavirenz (EFV)*	11(100.0)	K103N, 4 (36.4)
Nevirapine (NVP)*	11 (100.0)	V106M, 1 (9.1)
Delavirdine (DLV)	11 (100.0)	Y181C/I/V, 3 (27.3)
Etravirine (ETV)	6 (54.5)	Y188L, 2 (18.2)
Rilpivirine (RPV)	8 (72.7)	G190A/Q, 2 (18.2)
		M230L, 2(18.2)
Nucleoside reverse transcriptase inhibitors (NRTI, any)	9 (81.8)	
Lamivudine (3TC)*	9 (81.8)	K65R, 3 (27.3)
Azidothymidine (AZT)*	0 (0)	D67N, 3 (27.3)
Stavudine (D4T)*	3 (27.2)	V75L, 1 (9.1)
Didanosine (DDI)*	5 (45.5)	T69N, 1 (9.1)
Abacavir (ABC)	9 (81.8)	M184V/I, 8 (72.7)
Emtricitabine (FTC)	9 (81.8)	
Tenofovir (TDF) *	4 (36.4)	
Protease inhibitors+ (PI, any)	0 (0.0)	
Multi-drug resistance to NNRTI and NRTI	9(81.8)	

*Provided through the National Free Antiretroviral Treatment Program (NFATP).

+ Protease inhibitors (PI):including ATV, DRV, FPV, IDV, LPV, NFV, SQV, and TPV.

The factors significantly associated with successful viral suppression at 12 month were analysed through univariable logistic regression (Table 3). HIV transmission route, CD4 count at baseline, and missed doses in the past month were independent risk factors associated with virological failure (HIV RNA ≥1000 copies/ml). These three factors remained in the multivariable logistic regression model. Injecting drug use [adjusted odds ratio (AOR) = 0.40,95% CI: 0.19,0.84; P = 0.0154], CD4 count at baseline ≥350 cells/mm^3^(AOR = 0.32,95% CI: 0.14,0.72;P = 0.0056), and missed doses in the past month(AOR = 0.30,95% CI: 0.15,0.60;P = 0.0006) were negatively associated with virological success.

**Table 3:** Factors associated with HIV RNA <1000 copies/ml among HIV patients receiving first-line ART at 12 months in four sentinel antiretroviral treatment sites.

Variable	Number	HIV RNA <1000copies/ml *N* (%)	Crude OR (95% CI)	*P-value*	Adjusted OR (95% CI)	*P-value*
Total	448	394 (87.9)				
Age (year)						
≤38	196	174 (88.8)				
>38	252	220 (87.3)	0.87(0.49,1.55)	0.6347		
Sex						
Male	345	301 (87.3)				
Female	103	93 (90.3)	1.34 (0.66,2.81)	0.4066		
Ethnicity						
Han	403	352 (87.3)				
Other	45	42 (93.3)	2.03(0.61,6.79)	0.2510		
Education						
Junior highschool or less	204	174 (85.3)				
High school or more	244	220 (90.2)	1.58 (0.89,2.80)	0.1170		
Marital status						
Married	248	219 (88.3)				
Other	200	175 (87.5)	0.93 (0.52,1.64)	0.7944		
Occupation						
Other	409	360 (88.0)				
Farmer	39	34 (87.2)	0.93 (0.35,2.48)	0.8777		
HIV transmission route						
Other	393	353 (89.8)				
Drug injection	55	41 (74.5)	0.33 (0.17,0.66)	0.0017	0.40 (0.19,0.84)	0.0154
WHO clinic stage at baseline						
I or II	284	249 (87.7)				
III or IV	164	145 (88.4)	1.07 (0.59,1.95)	0.8171		
HIV RNA at baseline						
<1000 copies/mL	28	26 (92.9)				
≥1000 copies/mL	420	368 (87.6)	0.54(0.13,2.36)	0.4166		
CD4 count at baseline (cells/mm^3^)						
0–349	408	364 (89.2)				
350 or above	40	30 (75.0)	0.36 (0.17,0.79)	0.0109	0.32 (0.14,0.72)	0.0056
Initial ART regimen						
3TC+TDF+NVP/EFV	40	34(85.0)				
AZT+3TC+NVP/EFV	339	299 (88.2)	1.32 (0.52,3.33)	0.5585		
D4T+3TC+NVP/EFV	69	61 (88.4)	1.35(0.43,4.20)	0.6091		
Switched to second-line regimens						
No	441	389(88.2)				
Yes	7	5(71.4)	0.33(0.06,1.77)	0.1969		
ART distribution institution						
County hospital or CDC	377	334 (88.6)				
Village clinic or township hospital	71	60 (84.5)	0.70 (0.34,1.44)	0.3333		
Interval of ART refills						
≤90 days	209	180 (86.1)				
>90 days	239	214 (89.5)	1.38 (0.78,2.44)	0.2694		
Missed doses in the past month						
No	385	349 (90.7)				
Yes	63	45(71.4)	0.26 (0.14,0.49)	<0.0001	0.30 (0.15,0.60)	0.0006
Ratio (95%) of on-time drug intake in the past month						
No	72	44 (61.1)				
Yes	376	350(93.1)	8.57 (4.61,15.9)	<0.0001		
Stopped the regimen in the past month						
No	440	394(89.6)				
Yes	8	0	<0.001	0.9814		

## Discussion

In this one-year prospective follow-up survey on HIVDR at sentinel sites in four Chinese provinces, viral load suppression (HIV RNA <1000 copies/mL)was achieved at 12 months in 87.9% of treated patients, with a range from 82.9% to 93.6%. This demonstrated a rapid but successful scale up of China’s NFATP. In the 2012 WHO drug resistance surveillance guideline, viral load suppression after 12 months of ART can be graded as poor (<70%), fair (70–85%), or excellent (>85%) [16]. All sentinel sites in our study met the excellent grade, and the overall suppression rate was comparable to treatment responses seen in other developing and developed countries [23]–[29]. As well, our previous surveillance studies in four and two sentinel sites in China showed that viral load suppression at 12 months was ≥70% and ≥85%, respectively [17], [18]. Together, these HIVDR sentinel surveys indicated that virological failure did not increase over time during recent years of the NFATP’s expansion, when approximately 50,000 ART-naïve HIV patients initiated treatment each year. Our findings also provide support to the argument that the current WHO target of >70% viral load suppression at 12 months of treatment for low- and middle-income countries can be revised upwards, to such a rate as 85% [16].

The drug resistance mutations identified concurred with expected mutation patterns from a treatment program based on NNRTI and NRTI class drugs. Among patients with virological failure, 11 cases (27.5%) displayed NNRTI mutations and 9 cases (22.5%) displayed NRTI mutations. No protease inhibitor (PI) mutations were identified, which affirms that the continued use of PIs as second-line therapy is still viable in China. Our HIVDR results are comparable to reported values in other developed and developing countries [23]–[29].

Our study found that injecting drug use, baseline CD4 count ≥350, and missed doses in the past month were independently associated with virological failure (HIV RNA ≥1000 copies/ml) among HIV patients receiving free first-line ART in China. Virological failure as a result of poor adherence indicates that strengthening education and counseling among HIV patients should be an important priority for HIVDR prevention in China [14], [30]. Training for health care providers should also be improved, especially among those with fewer resources to new treatment technology. Another concern was the high proportion of virological failure among injection drug users (IDUs) [17], [31]. Low uptake of MMT and needle exchange services and high syphilis incidence suggest that large numbers of IDUs engaged in high-risk practices and may have transmitted HIVDR to others [32]. Efforts should be made to scale up IDU services to enhance ART adherence, and harm reduction programs should be considered as an essential part of HIV treatment. Additional data is needed for understanding how the dual treatment systems for HIV and drug addiction can be successfully integrated to reduce death and transmission of blood infections.

Lastly, the association of higher CD4 cell count with virologic failure warrants further investigation. Recent WHO guideline changes and advocacy towards initiating ART treatment at higher CD4 levels (≤500 cells/mm^3^ or immediately after testing) [33], [34] have raised concerns for adherence in asymptomatic individuals. Evidence on this issue currently remains sparse internationally and has tended to show that early treatment leads to virological and clinical benefits [35], [36]. However, a large study in China has also shown that patients with higher CD4 cell counts (≥300 cells/mm^3^) at cART initiation were more likely to drop out of HIV care [37]. Here in our cohort, the rate of loss to follow-up (including transfers out of the study follow-up districts) among patients with CD4 cell counts ≥350 cells/mm^3^ and <350 cells/mm^3^were 8.9%(5/45)and 7.7%(36/468), respectively(p = 0.77). Although the difference is not statistically significant, the sample size of patients with higher CD4 cell count is small, and more data is needed to confirm the observed relationship. In general, further studies are needed to clarify the effects of early ART initiation on adherence and attrition at short-term versus long-term treatment timescales in China.

This study has some limitations. First, due to limited geographic distribution of the sentinel sites and participant exclusion criteria, these results may not be fully representative of all of China. Second, the overall retention rate at 12 months was 87.3%, but only 79.0% in Hunan province; results should therefore be interpreted with caution. Third, due to a relatively low rate of successful HIVDR sequencing, the HIVDR rate from this study is likely lower than the true drug resistance rate. Finally, a 12-month follow up survey is relatively short for the purposes of monitoring HIVDR, and more studies with long-term monitoring is recommended to provide more objective and accurate data on viral suppression, HIVDR incidence, and optimal choices of regimen.

In sum, this HIVDR surveillance study in four sentinel sites across China demonstrates good virological and immunological outcomes at 12 months among these who initiated first-line ART treatment. However, patients infected through drug injection, who missed doses, or with higher CD4 at baseline are at increased risk for poor virological response. More data from these high-risk patients are needed to evaluate clinical outcomes and long-term effects of treatment.

## References

[1] Staszewski S, Morales-Ramirez J, Tashima KT, Rachlis A, Skiest D, et al (1999) Efavirenz plus zidovudine and lamivudine, efavirenz plus indinavir, and indinavir plus zidovudine and lamivudine in the treatment of HIV-1 infection in adults. Study 006 Team. N Engl J Med 341: 1865–1873. 10601505 10.1056/NEJM199912163412501

[2] MacArthur RD, Novak RM, Peng G, Chen L, Xiang Y, et al (2006) A comparison of three highly active antiretroviral treatment strategies consisting of non-nucleoside reverse transcriptase inhibitors, protease inhibitors, or both in the presence of nucleoside reverse transcriptase inhibitors as initial therapy (CPCRA 058 FIRST Study): a long-term randomised trial. Lancet 368: 2125–2135. 17174704 10.1016/S0140-6736(06)69861-9

[3] Gabillard D, Lewden C, Ndoye I, Moh R, Segeral O, et al (2013) Mortality, AIDS-morbidity, and loss to follow-up by current CD4 cell count among HIV-1-infected adults receiving antiretroviral therapy in Africa and Asia: data from the ANRS 12222 collaboration. J Acquir Immune Defic Syndr 62: 555–561. 23274931 10.1097/QAI.0b013e3182821821 PMC3921662

[4] Lima VD, Hogg RS, Harrigan PR, Moore D, Yip B, et al (2007) Continued improvement in survival among HIV-infected individuals with newer forms of highly active antiretroviral therapy. AIDS 21: 685–692. 17413689 10.1097/QAD.0b013e32802ef30c

[5] Borrell C, Rodriguez-Sanz M, Pasarin MI, Brugal MT, Garcia-de-Olalla P, et al (2006) AIDS mortality before and after the introduction of highly active antiretroviral therapy: does it vary with socioeconomic group in a country with a National Health System? Eur J Public Health 16: 601–608. 16698886 10.1093/eurpub/ckl062

[6] Joint United Nations Programme on HIV/AIDS. Together we will end AIDS. (Update 2012). Available: http://www.unaids.org/en/media/unaids/contentassets/documents/epidemiology/2012/20120718_ togetherwewillendaids_en.pdf. Accessed 2012 Jul 19.

[7] Chinese Center for Disease Control and Prevention: Analysis of HIV/STD epidemic in 2012. Beijing, China.

[8] Zhang F, Dou Z, Ma Y, Zhang Y, Zhao Y, et al (2011) Effect of earlier initiation of antiretroviral treatment and increased treatment coverage on HIV-related mortality in China: a national observational cohort study. Lancet Infect Dis 11: 516–524. 21600849 10.1016/S1473-3099(11)70097-4

[9] Zhang F, Dou Z, Ma Y, Zhao Y, Liu Z, et al.. (2009) Five-year outcomes of the China National Free Antiretroviral Treatment Program. Ann Intern Med 151: 241–251, W-252. 10.7326/0003-4819-151-4-200908180-00006 19687491

[10] Zhang F, Dou Z, Yu L, Xu J, Jiao JH, et al (2008) The effect of highly active antiretroviral therapy on mortality among HIV-infected former plasma donors in China. Clin Infect Dis 47: 825–833. 18690805 10.1086/590945 PMC2538607

[11] Liao L, Xing H, Su B, Wang Z, Ruan Y, et al (2013) Impact of HIV drug resistance on virologic and immunologic failure and mortality in a cohort of patients on antiretroviral therapy in China. AIDS 27: 1815–1824. 23803794 10.1097/QAD.0b013e3283611931 PMC3694318

[12] Xing H, Ruan Y, Li J, Shang H, Zhong P, et al (2013) HIV drug resistance and its impact on antiretroviral therapy in Chinese HIV-infected patients. PLoS One 8: e54917. 23405098 10.1371/journal.pone.0054917 PMC3566114

[13] Xing H, Wang X, Liao L, Ma Y, Su B, et al (2013) Incidence and associated factors of HIV drug resistance in Chinese HIV-infected patients receiving antiretroviral treatment. PLoS One 8: e62408. 23638072 10.1371/journal.pone.0062408 PMC3640055

[14] Wang X, He C, Xing H, Liao L, Xu X, et al (2012) Short communication: emerging transmitted HIV type 1 drug resistance mutations among patients prior to start of first-line antiretroviral therapy in middle and low prevalence sites in China. AIDS Res Hum Retroviruses 28: 1637–1639. 22822770 10.1089/aid.2012.0164 PMC3505058

[15] Liao L, Xing H, Dong Y, Qin G, Ma Y, et al (2012) Surveys of transmitted HIV drug resistance in 7 geographic Regions in China, 2008–2009. Clin Infect Dis 54 Suppl 4S320–323. 22544196 10.1093/cid/cir1016

[16] Protocol for population-based monitoring of HIVDR emerging during treatment and related program factors at sentinel ART clinics: 2012 update. Available: http://apps.who.int/iris/bitstream/10665/75205/1/WHO_HIV_2012.15_eng.pdf. Accessed 2013 Jun 16.

[17] Wang X, Yang L, Li H, Zuo L, Liang S, et al (2011) Factors associated with HIV virologic failure among patients on HAART for one year at three sentinel surveillance sites in China. Curr HIV Res 9: 103–111. 21361864 10.2174/157016211795569122

[18] Ruan Y, Xing H, Wang X, Tang H, Wang Z, et al (2010) Virologic outcomes of first-line HAART and associated factors among Chinese patients with HIV in three sentinel antiretroviral treatment sites. Trop Med Int Health 15: 1357–1363. 20868414 10.1111/j.1365-3156.2010.02621.x

[19] Manual of the National Free Antiretroviral Treatment, third edition. Available: http://www.chinaaids.cn/jszn/201301/t20130111_75763.htm.pdf. Accessed 2013 Jun 12.

[20] Shafer RW, Rhee SY, Pillay D, Miller V, Sandstrom P, et al (2007) HIV-1 protease and reverse transcriptase mutations for drug resistance surveillance. AIDS 21: 215–223. 17197813 10.1097/QAD.0b013e328011e691 PMC2573394

[21] Liu TF, Shafer RW (2006) Web resources for HIV type 1 genotypic-resistance test interpretation. Clin Infect Dis 42: 1608–1618. 16652319 10.1086/503914 PMC2547473

[22] Zhong P, Pan Q, Ning Z, Xue Y, Gong J, et al (2007) Genetic diversity and drug resistance of human immunodeficiency virus type 1 (HIV-1) strains circulating in Shanghai. AIDS Res Hum Retroviruses 23: 847–856. 17678466 10.1089/aid.2006.0196

[23] McMahon JH, Elliott JH, Bertagnolio S, Kubiak R, Jordan MR (2013) Viral suppression after 12 months of antiretroviral therapy in low- and middle-income countries: a systematic review. Bull World Health Organ 91: 377–385E. 23678201 10.2471/BLT.12.112946 PMC3646348

[24] Wadonda-Kabondo N, Bennett D, van Oosterhout JJ, Moyo K, Hosseinipour M, et al (2012) Prevalence of HIV drug resistance before and 1 year after treatment initiation in 4 sites in the Malawi antiretroviral treatment program. Clin Infect Dis 54 Suppl 4S362–368. 22544204 10.1093/cid/cir987 PMC3338306

[25] Laurent C, Diakhate N, Gueye NF, Toure MA, Sow PS, et al (2002) The Senegalese government’s highly active antiretroviral therapy initiative: an 18-month follow-up study. AIDS 16: 1363–1370. 12131213 10.1097/00002030-200207050-00008

[26] Koenig SP, Leandre F, Farmer PE (2004) Scaling-up HIV treatment programmes in resource-limited settings: the rural Haiti experience. AIDS 18 Suppl 3S21–25. 15322480 10.1097/00002030-200406003-00005

[27] Smith D, Berrey MM, Robertson M, Mehrotra D, Markowitz M, et al (2000) Virological and immunological effects of combination antiretroviral therapy with zidovudine, lamivudine, and indinavir during primary human immunodeficiency virus type 1 infection. J Infect Dis 182: 950–954. 10950796 10.1086/315753

[28] Boileau C, Nguyen VK, Sylla M, Machouf N, Chamberland A, et al (2008) Low prevalence of detectable HIV plasma viremia in patients treated with antiretroviral therapy in Burkina Faso and Mali. J Acquir Immune Defic Syndr 48: 476–484. 18614917 10.1097/QAI.0b013e31817dc416

[29] Fielding KL, Charalambous S, Stenson AL, Pemba LF, Martin DJ, et al (2008) Risk factors for poor virological outcome at 12 months in a workplace-based antiretroviral therapy programme in South Africa: a cohort study. BMC Infect Dis 8: 93. 18631397 10.1186/1471-2334-8-93 PMC2494994

[30] Ma Y, Zhao D, Yu L, Bulterys M, Robinson ML, et al (2010) Predictors of virologic failure in HIV-1-infected adults receiving first-line antiretroviral therapy in 8 provinces in China. Clin Infect Dis 50: 264–271. 20017637 10.1086/649215 PMC2805417

[31] Rodriguez-Arenas MA, Jarrin I, del Amo J, Iribarren JA, Moreno S, et al (2006) Delay in the initiation of HAART, poorer virological response, and higher mortality among HIV-infected injecting drug users in Spain. AIDS Res Hum Retroviruses 22: 715–723. 16910826 10.1089/aid.2006.22.715

[32] Ruan Y, Liang S, Zhu J, Li X, Pan SW, et al (2013) Evaluation of harm reduction programs on seroincidence of HIV, hepatitis B and C, and syphilis among intravenous drug users in southwest China. Sex Transm Dis 40: 323–328. 23486498 10.1097/OLQ.0b013e31827fd4d4

[33] World Health Organization (WHO). Consolidated guidelines on the use of antiretroviral drugs for treating and preventing HIV infection. June 2013. Available: http://www.who.int/hiv/pub/guidelines/arv2013/en/index.html.pdf. Accessed 2013 Jul 11.

[34] Cohen MS, Chen YQ, McCauley M, Gamble T, Hosseinipour MC, et al (2011) Prevention of HIV-1 infection with early antiretroviral therapy. N Engl J Med 365: 493–505. 21767103 10.1056/NEJMoa1105243 PMC3200068

[35] Uy J, Armon C, Buchacz K, Wood K, Brooks JT, et al (2009) Initiation of HAART at higher CD4 cell counts is associated with a lower frequency of antiretroviral drug resistance mutations at virologic failure. J Acquir Immune Defic Syndr 51: 450–453. 19474757 10.1097/QAI.0b013e3181acb630

[36] Resistance UKCGoHD (2010) Group UCS (2010) Long-term probability of detecting drug-resistant HIV in treatment-naive patients initiating combination antiretroviral therapy. Clin Infect Dis 50: 1275–1285. 20353366 10.1086/651684

[37] Zhu H, Napravnik S, Eron J, Cole S, Ma Y, et al (2012) Attrition among human immunodeficiency virus (HIV)- infected patients initiating antiretroviral therapy in China, 2003–2010. PLoS One 7: e39414. 22761787 10.1371/journal.pone.0039414 PMC3384674

